# Prevalence and genetic characterization of *Cryptosporidium* species and *Giardia duodenalis* in lambs in Oromia Special Zone, Central Ethiopia

**DOI:** 10.1186/s12917-016-0916-0

**Published:** 2017-01-17

**Authors:** Teklu Wegayehu, Md Robiul Karim, Junqiang Li, Haileeyesus Adamu, Berhanu Erko, Longxian Zhang, Getachew Tilahun

**Affiliations:** 1 Aklilu Lemma Institute of Pathobiology, Addis Ababa University, Addis Ababa, Ethiopia; 2 College of Natural Sciences, Arba Minch University, Arba Minch, Ethiopia; 3 College of Animal Sciences and Veterinary Medicine, Henan Agricultural University, Zhengzhou, Henan China; 4 Faculty of Veterinary Medicine and Animal Science, Bangabandhu Sheikh Mujibur Rahman Agricultural University, Gazipur, 1706 Bangladesh; 5 Institute of Biotechnology, Addis Ababa University, Addis Ababa, Ethiopia

**Keywords:** *Cryptosporidium*, *Giardia duodenalis*, Zoonotic transmission, Lamb, Ethiopia

## Abstract

**Background:**

*Cryptosporidium* and *Giardia duodenalis* are gastro-intestinal parasites that infect human and animals worldwide. Both parasites share a broad host range and are believed to be zoonosis. The aim of this study was to identify the species of *Cryptosporidium* and assemblages of *G. duodenalis* in lambs and to elucidate their role in zoonotic transmission.

**Results:**

A total of 389 fecal samples were collected from lambs and screened by microscopy and nested PCR targeting the small-subunit ribosomal RNA for *Cryptosporidium;* and the small-subunit ribosomal RNA, triose phosphate isomerase, β-giardin*,* and glutamate dehydrogenase genes for *G. duodenalis.* The prevalence of *Cryptosporidium* and *G. duodenalis* was 2.1% (8/389) and 2.6% (10/389), respectively. The infection rate at the three study sites ranged from 1.3 to 3.1% for *Cryptosporidium* and 1.6 to 3.9% for *G. duodenalis*; but variation was not statistically significant (*p* > 0.05). The finding also showed that there is no sex and age group associated difference in the occurrence of *Cryptosporidium* and *G. duodenalis* infections in lambs. Sequence analysis revealed that lambs were mono-infection with *C. ubiquitum* and *G. duodenalis* assemblage E. The analysis also indicated the presence of genetic variation within isolates of assemblage E; with 4 of them are novel genotypes at the small-subunit ribosomal RNA, β-giardin*,* and glutamate dehydrogenase genes.

**Conclusion:**

The findings of the current study showed that lambs are capable of harboring *C. ubiquitum* and *G. duodenalis* assemblage E. This finding suggests that lambs might be sources for potentially zoonotic *Cryptosporidium* species. This was first molecular study in lambs and contributes to a better understanding of the epidemiology of *Cryptosporidium* and *G. duodenalis* in central Ethiopia.

## Background

*Cryptosporidium* and *Giardia* are protozoan parasites that cause diarrhea in humans and animals worldwide [1]. These parasites are transmitted via the fecal-oral route following contact with the infective stages of the parasites. Although the question of zoonotic transmission is clearer for *Cryptosporidium* than *Giardia,* both share a wide host range and are thought to be zoonosis [2]. Livestock have taken part in the disease cycle, and have been identified as the sources of numerous outbreaks of human cryptosporidiosis and giardiasis [3].

There is considerable genetic diversity within *Cryptosporidium* and *Giardia duodenalis*. Up till now, 26 *Cryptosporidium* species and more than 70 genotypes have been recognized [4]. *G. duodenalis* (syn. *G. lamblia* and *G. intestinalis*) is also considered a multispecies complex, with at least eight distinct genetic groups or assemblages (A–H) [5]. Assemblages A and B in humans and animals; assemblages C and D in dogs, assemblage E in cattle, sheep and pigs, assemblage F in cats, assemblage G in rats and assemblage H in seals have been described [1, 5].

*Cryptosporidium* and *G. duodenalis* infections in sheep are fairly common and have been reported globally. Genetic characterization of *Cryptosporidium* in sheep has demonstrated the occurrence of the zoonotic species *C. parvum* and *C. ubiquitum*; and the host-adapted species *C. bovis*, *C. xiaoi* and *C. andersoni* [6–11]. Three assemblages of *G. duodenalis* have been recognized in sheep, assemblage E, and the two zoonotic assemblages, assemblages A and B [6, 7, 12, 13]. These studies have provided the evidence that sheep may harbor species of *Cryptosporidium* and genotypes/assemblages of *G. duodenalis* which are potentially infectious to humans.

Reports from different parts of Ethiopia have showed different prevalence rate of cryptosporidiosis and giardiasis in humans [14–17]. Recent study conducted on giardiasis showed prevalence of 16.8% in children in Holetta, Sendafa and Chancho [18]. In addition, molecular studies conducted in humans identified *C. parvum* zoonotic subtype family IIa [19, 20] and *G. duodenalis* assemblage A and B [18, 21, 22] as the major cause of human cryptosporidiosis and giardiasis in Ethiopia. However, no molecular studies have conducted in sheep to assess the prevalence and genotypes that they harbor. Therefore, the aim of the present study was to identify the species of *Cryptosporidium* and assemblages of *G. duodenalis* infection in lambs using DNA sequence analysis to assess their role in zoonotic cycle of the diseases.

## Methods

### Study area and study animal

This cross-sectional study was conducted between January and June 2014 in three areas of Oromia Special Zone located within 40 km radius of the capital city, Addis Ababa. Based on the available climatological data, the mean annual rainfall of the Special Zone varies from 700 mm to 1400 mm in lowlands and highlands, respectively. The mean annual temperature of the Zone ranges between 20 and 25 °C in the lowlands and 10 to15 °C in the central highlands.

In Ethiopia, small ruminants are important components of the livestock subsector and kept as source of family income, meat, milk, food and wool by small holder farmers throughout the country. It is estimated that about 1,078,000 sheep are annually used for domestic meat consumption. In this study, lambs were included from three districts, Holetta, Sendafa and Chancho town.

### Specimen collection

A total of 389 fecal samples were collected from lambs age younger than 5 months in separate and labeled stool containers. The specimens were taken either directly from the rectum of each lamb or from the ground immediately after defecation using sterile disposable gloves. During sample collection, the identification number, age and sex of each lamb were recorded. A part of the samples was used for microscopy and the remaining were preserved in 2.5% potassium dichromate in 1:1 ratio and stored at 4 °C before Deoxyribonucleic acid (DNA) extraction.

### Microscopy

Fresh stool sample was tested using light microscope to detect trophozoites and cysts of *G. duodenalis* using the Lugol’s iodine staining at 10X and 40X magnifications. Thin smears were prepared from sediments of formol-ether concentrated stool samples. The smears were stained with modified Ziehl-Neelson staining method [23]. In brief, air-dried thin smears were fixed with absolute methanol for 5 min, air-dried and stained with carbol-fuchsin for 30 min. Smears were washed with tap water and decolorized with 1% acid-alcohol for 2 min. It was washed with tap water and counterstained with 1% methylene blue for another 2 min, rinsed again in tap water and air-dried. Finally, the stained smears were examined at 100X magnification to detect oocysts of *Cryptosporidium*.

### DNA extraction

After washing the preserved fecal specimens with deionized water, the genomic DNA was extracted from each fecal sample using the E.Z.N.A.® Stool DNA kit (Omega Biotek Inc., Norcross, USA). Concisely, about 50–100 mg of fecal specimen was added in a 2 ml centrifuge tube containing 200 mg of glass beads and placed on ice. Following, 300 μl buffer SP1 and proteinase K were added, and incubated at 70 °C for 10 min. Subsequently, all the procedures outlined in product manual were performed according to the manufacturer’s protocol. Finally, DNA was eluted in 200 μl elution buffer and the extract was stored at −20 °C until used in PCR.

### Nested PCR

Approximately 830 bp fragment of the small-subunit (*ssu*) ribosomal RNA gene of *Cryptosporidium* was amplified using nested PCR as described [24]. The amplification was done in 25 μl reaction volume containing 24 μl mixes and 1 μl DNA template. The PCR was performed using Applied Biosystems Thermal Cycler version 2.09. The reaction mixture was initially incubated at 94 °C for 5 min for denaturation and then a total 35 cycles of reaction were performed for final denaturation at 94 °C for 45 s, primer annealing at 55 °C for 45 s and strand extension at 72 °C for 1 min. The final extension was done at 72 °C for 10 min and cooled at 4 °C.

The nested PCR of *G. duodenalis* was conducted by amplification of the *ssu* rRNA, triose phosphate isomerase *(tpi)*, β-giardin *(bg),* and glutamate dehydrogenase *(gdh)* genes. Previously described mixes and PCR conditions were used to amplify fragments of the *ssu rRNA* gene [25], the *bg* gene [26], the *gdh* gene [27] and the *tpi* gene [28], with some modifications. The primers used, their amplicon size, annealing temperature, and the main uses of the targeted genes are described [29].

The secondary PCR was run under the same reaction and cycling conditions as the primary PCR, except the amplicon of the primary PCR was used as a template. The secondary amplified products were separated by electrophoresis on 1% agarose gel and visualized under a trans-illuminator after staining with ethidium bromide.

### DNA sequence analysis

The PCR products purified using Montage PCR filters (Millipore, Bedford, MA) were sequenced in both directions using forward and reverse primers with an ABI BigDye Terminator v. 3.1 cycle sequencing kit (Applied Biosystems, Foster City, CA) on an ABI 3100 automated sequencer. The sequence and chromatograms obtained from each strand were analyzed using ClustalX software. Consensus sequences were then compared with sequences in GenBank database using the Basic Local Alignment Search Tool (BLAST) (http://www.ncbi.nlm.nih.gov/blast/) to identify the species of *Cryptosporidium* and assemblages of *G. duodenalis*.

### Statistical analysis

Data were entered into the compute by EpiData version 3.1 and analysed using STATA software. Chi square test was used to verify possible association between infections with the parasites and different factors. Values were considered to be statistically significant when the *p* value was less than 0.05.

## Results

From the total of 389 lambs, 103, 158 and 128 were sampled from Holetta, Sendafa and Chancho areas, respectively. Of these, 201 were female and 188 were male lambs with female to male ratio of 1:0.9 (Table 1). The mean age of the lambs were 2.1 (ranged: 0.1 to 5 months). The study lambs were apparently healthy and no symptom of disease was observed during sample collection.

**Table 1:** Prevalence of *Cryptosporidium* species and *G. duodenalis* in lambs by study site, sex and age group in Oromia Special Zone, central Ethiopia (January–June, 2014)

Parameters	Samples examined (*N* = 389)	Parasites studied					
		*Cryptosporidium*			*G. duodenalis*		
		No. of samples positives n (%)	*χ*2	*p* value	No. of samples positives n (%)	*χ*2	*p* value
Study Site							
Holetta	103	2 (1.9)			4 (3.9)		
Sendafa	158	2 (1.3)	5.285	0.071	4 (2.5)	1.229	0.542
Chancho	128	4 (3.1)			2 (1.6)		
Sex							
Male	188	3 (1.6)	0.383	0.536	2 (1.1)	3.298	0.069
Female	201	5 (2.5)			8 (4.0)		
Age group							
<5 weeks	90	3 (3.3)			0 (0.0)		
5–8 weeks	163	3 (1.8)	0.998	0.607	5 (3.1)	3.199	0.202
>8 weeks	136	2 (1.5)			5 (3.7)		

Key: *χ*2 and *p* values compare the prevalence between sex, age and breed groups in calves

### Prevalence of *Cryptosporidium* and *G. duodenalis* infections

**Fig. 1:**
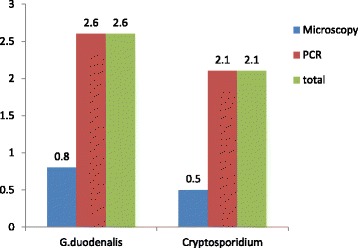
Prevalence of *Cryptosporidium* and *G. duodenalis* infection in lambs (*n* = 389) by microscopy and PCR

The overall prevalence of *Cryptospodidium* and *G. duodenalis* infection in lambs based on microscopy and nested PCR was 2.1% (8/389) and 2.6% (10/389), respectively (Fig. 1). The infection rate at the three study sites ranged from 1.3 to 3.1% for *Cryptosporidium* and 1.6 to 3.9% for *G. duodenalis*. The variation was statistically not significant (*p* > 0.05) between the study sites (Table 1). The finding also showed that there is no sex and age group associated difference in the occurrence of *Cryptosporidium* and *G. duodenalis* infections in lambs.

### Molecular characterization

Eight isolates of *Cryptosporidium* identified from lambs by PCR amplification of *ssu* rRNA gene were successfully sequenced. Sequence analysis showed that all the isolates belonged to *C. ubiquitum*. Comparison with *ssu* rRNA sequences available in the GenBank shown 100% sequence identity with sequences of isolates previously recognized from sheep, lemurs, wildlife and storm water (accession numbers: EU827398, AF442484, EF641018 and AY737592).

The multi-locus analysis of *G. duodenalis* obtained from lamb specimens are shown in Table 2. Among the 10 positive specimens, 5 were successfully amplified and sequenced at *tpi* locus only. The remaining positive specimens were identified by the genes in common. Sequence analysis of the isolates at the *ssu* rRNA, *tpi, bg* and *gdh*, genes revealed the presence of *G. duodenalis* assemblage E.

**Table 2:** Assemblages of *G. duodenalis* as determined by sequence analysis of *ssu* rRNA*, tpi bg* and *gdh* genes in lambs

Study site	Isolate code	Lambs		Assemblages by thte four genes			
		Sex	Age	*ssu* rRNA	*tpi*	*bg*	*gdh*
Holetta	HL-45	F	2	**-**	E	**-**	
	HL-51	M	3.5	E*	**-**	**-**	E*
	HL-87	F	4	E*	**-**	**-**	E*
	HL-91	F	3	E*	**-**	E	**-**
Sendafa	SL-12	F	3	**-**	E	**-**	**-**
	SL-86	M	2	**-**	E	E*	**-**
Chancho	CL-152	F	1.5	**-**	E	**-**	**-**
	CL-169	F	3.5	**-**	E	**-**	**-**
	DL-07	M	2	**-**	E	**-**	**-**
	AL-02	F	3	**-**	E	E*	**-**

Key: Asterisks (*) indicate novel genotypes; hyphens (-) indicate PCR-negative results

BLAST analysis of sequences obtained from the three isolates at the *ssu* rRNA gene showed 100% similarity to each other, but had 99% similarity to sequence registered in the GenBank (accession number AY655701). They had one additional nucleotide (G) at position 271, and named as ET-L1.

The analysis of the 7 isolates at the *tpi* gene revealed the presence of three distinct genotypes, named as E1, E2 and E3, for a convenient description (Table 3). These isolates showed 100% sequence identity to assemblage E identified from cattle, sheep and goat kids with GenBank accession numbers: KJ363351, JF792419 and EU189333, respectively. Isolates E2 and E3 had 2–3 SNPs compared to the reference (JF792419).

**Table 3:** Genetic variants within sub-genotypes of *G. duodenalis* assemblage E at the *tpi, bg* and *gdh* gene in lambs

Genetic variants	GenBank accession no	No. of isolates	Nucleotide at positions								
			36	50	57	78	131	144	200	254	512
*tpi gene*											
E (Ref.)	JF792419		G	A	C	C	C	A	A	A	G
E1	KT922261	4	*	*	*	*	*	*	*	*	*
E2	KT922262	2	A	*	T	*	*	G	*	*	*
E3	KT922260	1	*	*	T	T	*	*	*	*	*
*bg gene*											
E (Ref.)	EU189361		G	C	G	A	G	T	A	A	G
E4	KT922250	1	*	*	*	*	*	*	*	*	*
ET-L2	KT922251	2	*	*	*	*	*	*	*	G	*
*gdh* gene											
E (Ref.)	KR048474		G	C	G	T	**G**	G	G	C	T
ET-L3	KT922256	1	*	*	*	*	*	*	A	*	*
ET-L4	KU196418	1	*	T	*	*	A	*	*	*	C

Key: Asterisks (*) represent nucleotide identity

Isolate with accession number KT922250 identified at the *bg* gene showed 100% similarity with assemblage E (accession numbers: EU189361, DQ116625, KP334150, and KC960639). The other isolates were novel (named as ET-L2); and showed 99% similarity to isolate with accession number EU189361. It had substitution of one nucleotide (A-to-G) at nucleotide position 254 (Table 3).

Analysis of the two lamb isolates identified at the *gdh* gene revealed the presence of two genotypes (Table 3). When these sequences were compared by BLAST analysis of sequences in the GenBank database, they revealed 99% nucleotide sequence uniqueness to the assemblage E registered in the database, and named as ET-L3 and ET-L4. Multiple alignments of these sequences with the reference sequence (KR048474) showed substitution at 50, 131, 200 and 512.

### Phylogenetic analysis

**Fig. 2:**
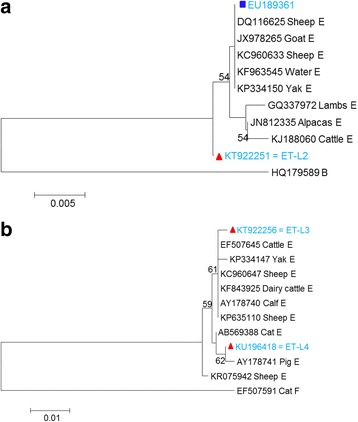
Phylogenetic tree based on nucleotide sequences of the *bg* gene (**a**) and *gdh* gene (**b**) of *G. duodenalis*. Trees were constructed using the neighbor-joining method based on genetic distance calculated by the Kimura 2-parameter model, employed in MEGA version 5.2. Bootstrap values >50% from 1,000 replicates is shown on nodes. Isolates showed known and novel sequences obtained from this study are marked by rectangle (*blue*) and triangles (*red*), respectively

Phylogenetic analysis was conducted using sequences obtained in this study at the *bg* and *gdh* genes and those available in GenBank database to clarify the genetic relationship among the genotypes. The novel genotype at the *bg* gene, ET-L2 (KT922251), had formed separate cluster from the reference sequences; whereas the known genotype with accession number EU189361 was clustered with the reference sequences (Fig. 2a). Analysis of one of the two new genotypes obtained at *gdh* gene, ET-L3 (KT922256), was clustered with reference sequences of assemblage E with accession numbers: EF507645, KP334147, KC960647, KF843925, AY178740 and KP635110. On the other hand, the second new genotype ET-L4 (KU196418) was clustered with sequence obtained from pig (accession number AY178741) (Fig. 2b).

## Discussion

*Cryptosporidium* species and *G. duodenalis* infections are relatively common and have been reported worldwide in sheep [6, 8, 13, 30, 31]. Although some reports suggest that sheep are probably not an important reservoirs for zoonotic *Cryptosporidium* species and *G. duodenalis* [6, 8], molecular characterization of the two parasites indicated that they can harbor species or genotypes/assemblages which are potentially infectious to humans [7, 8, 11–13, 30, 31].

The present study is the first to report the occurrence and genetic characterization of *Cryptosporidium* and *G. duodenalis* in sheep in Ethiopia. The overall infection rates were 2.1 and 2.6% for *Cryptospodidium* species and *G. duodenalis*, respectively, which was much lower than those reported from other parts of the world [6, 8, 12, 30, 31]. Even though *Cryptosporidium* is thought to be more prevalent in sheep than *G. duodenalis* [11]*,* the present study suggests that this may not always be the case, as the prevalence of these protozoan infections was not significantly different. This situation was also supported by report from Greece, where the prevalence of *Cryptosporidium* infection was much lower both in lambs and goat kids than the anticipated as compared to *G. duodenalis* [31].

Results of the present molecular analyses reveal that *C. ubiquitum* is the only species identified in lambs. Likewise, studies conducted in various parts of the world have identified *C. ubiquitum* as the common and the most prevalent species in sheep observed in different magnitude [6–8, 10–12, 30]. However, several studies have also reported species of *Cryptosporidium* other than *C. ubiquitum* [8, 10, 11, 30]. The zoonotic species, *C. parvum,* was not detected in lambs in the present study which is similar with the previous report [6]. This finding suggests that lambs may not be an important reservoir for *C. parvum* in Ethiopia*.*

*C. ubiquitum* has been reported in a wide variety of hosts including humans. The first report of *C. ubiquitum* in humans was found in fecal samples from patients with clinical symptoms of cryptosporidiosis [32]. Another study conducted to assess the importance of dairy cattle as a source of human *Cryptosporidium* infections in Ontario, Canada has also been reported *C. ubiquitum* in humans [33]. Subsequently, sporadic cases of this species affecting humans have been described [34, 35]. Therefore, *C. ubiquitum* should be considered a potential emerging zoonotic pathogen, and lambs can be considered as an important reservoir for this species in Ethiopia.

Sequence analysis of 10 *G. duodenalis* isolates revealed mono-infection with *G. duodenalis* assemblage E at the four loci, which is similar with the previous report [31]. This assemblage is usually found in hoofed animals including cattle and sheep [6–8, 10–12, 30], although the zoonotic genotypes A and B have also been observed in sheep in other studies [6, 8, 12, 13]. This observation indicates that lambs may not be an important reservoir for zoonotic assemblages of *G. duodenalis* in Ethiopia*.* However, further investigations are needed if this observation holds true in other parts of the country preferably involving larger sample size for a better understanding of epidemiology of cryptosporidiosis and giardiasis in lambs.

Molecular and phylogenetic analysis indicated that there is genetic variation within genotype E. Earlier allozyme data based on 23 allozyme loci shown that genotype E comprised three clusters of isolates; a pig cluster, a sheep cluster and a cattle cluster [36]. In the present study, all the sheep-derived isolates were showed clear clustering of isolates into host groups except one separate cluster in assemblage E. This is based on phylogenetic analysis of sequence data from two loci, *bg* and *gdh*.

## Conclusion

The present study demonstrates that lambs are capable of harboring *C. ubiquitum* and *G. duodenalis* assemblage E. These finding indicate that lambs may be important sources of potentially zoonotic *C. ubiquitum* but are not sources of zoonotic assemblages of *G. duodenalis* in the study localities. This is the first report of *Cryptosporidium* and *G. duodenalis* infection in lambs, and the obtained result would serve as a baseline data for further investigation for better understanding of the epidemiology of cryptosporidiosis and giardiasis in Ethiopia.
